# Mobile Robot Application with Hierarchical Start Position DQN

**DOI:** 10.1155/2022/4115767

**Published:** 2022-09-05

**Authors:** Emre Erkan, Muhammet Ali Arserim

**Affiliations:** ^1^Department of Electronic Communication, Batman University, Batman 72500, Turkey; ^2^Electrical and Electronics Engineering Department, Dicle University, Diyarbakir 21280, Turkey

## Abstract

Advances in deep learning significantly affect reinforcement learning, which results in the emergence of Deep RL (DRL). DRL does not need a data set and has the potential beyond the performance of human experts, resulting in significant developments in the field of artificial intelligence. However, because a DRL agent has to interact with the environment a lot while it is trained, it is difficult to be trained directly in the real environment due to the long training time, high cost, and possible material damage. Therefore, most or all of the training of DRL agents for real-world applications is conducted in virtual environments. This study focused on the difficulty in a mobile robot to reach its target by making a path plan in a real-world environment. The Minimalistic Gridworld virtual environment has been used for training the DRL agent, and to our knowledge, we have implemented the first real-world implementation for this environment. A DRL algorithm with higher performance than the classical Deep Q-network algorithm was created with the expanded environment. A mobile robot was designed for use in a real-world application. To match the virtual environment with the real environment, algorithms that can detect the position of the mobile robot and the target, as well as the rotation of the mobile robot, were created. As a result, a DRL-based mobile robot was developed that uses only the top view of the environment and can reach its target regardless of its initial position and rotation.

## 1. Introduction

The path planning is one of the important topics of the robotics since navigating a robot to a desired destination without collision is a significant task [1]. The significance of such a task is estimated to increase even further [2, 3] due to evolving interaction between robots and human beings. This means the human beings will have more places shared with robots giving rise to the importance of path planning. In literature, conventional path planning techniques such as A-star, ant colony optimization, and Artificial Potential Field (APF) can be encountered [4] which are not efficient to address the issues in real-time within complex environments [5]. With respect to achieving a more robust solution, deep learning (DL) has been successfully implemented for path planning [6]. However, DL needs labeled data in order to learn the environment which is a time-consuming task [7]. To overcome the latter problem, the researchers have recently adopted the deep reinforcement learning (DRL) technique as one of the efficient solutions.

Reinforcement learning (RL) tries to maximize the numerical reward signals by focusing on actions that must be taken depending on the specific cases [8]. Instead of guiding the RL agent to perform a specific task, it is asked to learn which actions are more rewarding [9]. The RL agent, interacting with the environment sufficiently, learns which action contributes more to the cumulative reward. In another word, it learns the best state-action pair. However, exploring the environment becomes costly in the case of increasing numbers of states and actions due to the curse of dimensionality [10]. Therefore, it may become impossible to solve some of the real-world problems with high dimensions of action and state spaces by using traditional reinforcement learning [11].

The remarkable progress of the DL has also significantly affected the RL and resulted in the DRL method, which is a combination of both methods [12]. Significant achievements have been reported for arcade games [13, 14], board games [15, 16], autonomous driving [17], robots interacting with humans [18], drones [19], unmanned surface vehicles [20], robot navigation problems [21], and robotic surgery [22], which all are examples of complex systems with multiple states.

Several recent studies on path planning of mobile robots by using the DRL method are briefly provided below:

- Zheng et al. [1] proposed a method that optimizes the path planning process of a mobile robot for an unmapped environment. In the proposed method, a memory module based on the special structure of long short-term memory (LSTM) was introduced. Thanks to this method, the long-term memory capacity of the robot was increased, and the number of trials and calculation time required for training was shortened.
- Gong et al. [5] suggested optimizing the deep deterministic policy gradient (DDPG) network with LSTM to solve the path planning problem of the mobile robot in environments with static obstacles. In this method, a mixed noise along with a more reasonable reward function was used for quick training. The proposed algorithm and DDPG-based algorithms [23, 24] were experimentally compared and the advantages of the proposed algorithm were demonstrated in a complex environment in terms of exploration efficiency, optimum path and time.
- In Zhou et al. [7], an improved DQN algorithm was proposed for the path planning problem of patrolling robots. In this study, the reward penalty functions were improved, and the sparse reward problem was resolved by optimizing the state-action space by adding new reward value points. The advantages of the proposed algorithm over the classical DQN algorithm were experimentally demonstrated.
- In Wang et al. [25], an improved DQN method was proposed by focusing on the problem of poor exploration and sparse reward in mobile robot path planning. The reward function was improved by combining the artificial potential field method with the reward function to optimize the state-action space. The performances of the proposed method and classical DQN were compared.
- In Xing et al. [26], the area division Deep Q-Network (AD-DQN) method was proposed. A mobile wireless powertrain robot was able to determine the optimal path with the proposed method in terms of charging a large number of IoT devices.
- Huang et al. [27] proposed a method that determines two reward thresholds for solving the anomalous reward problem encountered in the path planning process of a mobile robot in an unknown dynamic environment. The improvement in value-based DRL algorithms was experimentally demonstrated with the proposed method.
- Yu et al. [28] proposed a mobile robot path planning method based on neural networks and hierarchical reinforcement learning. The performance of the proposed method was evaluated for an environment with static obstacles.
- Wang et al. [29] proposed a DDQN-based method with prioritized experience replay (PER) for mobile robot path planning. Simulation experiments were shown that this method had a better convergence rate and success rate than classical DQN in unknown environments.
- Zhang et al. [30] focused on similar sample redundancy problems that negatively affect the training of the DQN algorithm for environments with static obstacles. An algorithm was proposed in this study in order to optimize the training samples using similarity scanning matrix such that more useful training examples can be used.
- Ruan et al. [31] proposed a D3QN-based LN-D3QN algorithm for mobile robots that avoid the obstacles. The proposed algorithm accelerated the neural network training by normalizing the layers of the neural network. In addition, it was observed that the learning time was reduced by using prior experience repetition. Experiments showed that the robot trained with the LN-D3QN algorithm can adapt to unknown environments faster than that of the D3QN algorithm.
- Kim et al. [32] proposed a DQN method combined with a Gated Recurrent Unit (GRU) to solve the path planning problem of a mobile robot.

On the contrary to the above listed works, in this study, a DRL agent was trained in a discrete virtual environment with sparse rewards and focused on the real-world targeting problem of a mobile robot using these DRL network parameters. The contributions of this study can be listed as follows:

- An improved DQN algorithm is proposed for large environments with sparse rewards in order to increase the convergence rate and success rate according to the classical DQN algorithm.
- Network parameters of the agent trained in the Minigrid virtual environment were used for the first time in the real environment.
- A pixel-based image processing algorithm is proposed to detect the rotation of the mobile robot.
- The model trained in the virtual environment is used to provide a more efficient operation in the real environment.
- The proposed design allows the mobile robot to be used in swarm robotics and multi-agent systems with the advantage of low cost and long-range controllability from a central computer.

The rest of the article is organized as follows.  Section 2 introduces the virtual environment used in DRL agent training. In Section 3, the DQN algorithm with hierarchical starting position is presented as an efficient method, in terms of training time and successfulness rate, compared to the conventional DQN algorithm.  Section 4 describes the design and control of the mobile robot used in the real environment.  Section 5 explains how to pair a discrete virtual environment with a real-world environment and the mode of operation of the DRL-based mobile robot which can achieve its target regardless of its starting position and rotation.  Section 6 explains the implementation stage and presents the performance analyses. Finally, the paper is concluded in Section 7.

## 2. Structure of Virtual Environment

OpenAI Gym is a toolkit designed for RL research. Creating a common interface, OpenAI Gym contains many different tasks and environments [33]. This study uses one of the OpenAI Gym platforms, the Minimalistic Gridworld Environment (MiniGrid). The MiniGrid Environment platform has numerous environments with increasing difficulty levels [34].

The studies regarding natural language processing [35–37], reinforcement learning for environments with sparse reward signals [38–45] and hierarchical-based reinforcement learning in natural language processing [46] can be found for MiniGrid environment. But the studies were limited to the virtual environment. This study focuses on real environment application by using the agent trained in virtual environment.

**Figure 1:**
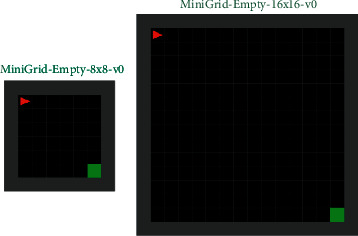
Environments to be used in the application.

The environments shown in Figure 1 are used in the application. Both environments represent an empty room, and the agent's goal is to reach the green target plot in an environment that provides sparse reward. The only difference between the two environments is the number of plots constituting the environment. Therefore, this section where virtual environment analysis is performed explains the structure of MiniGrid-Empty-8 × 8-v0 environment.

### 2.1. States

**Figure 2:**
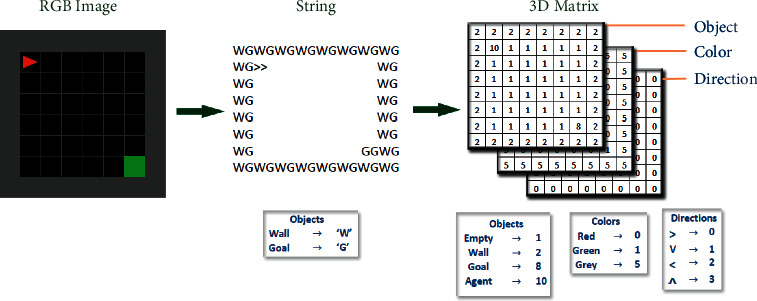
Virtual environment status obtained in three different ways.

The MiniGrid-Empty-8 × 8-v0 environment consists of 8 × 8 plots. The state of the virtual environment can be obtained in three different ways. As shown in Figure 2, these include;

- RGB image consisting of a combination of 32 pixels × 32 pixels plots by default,
- String structure in which each plot is encoded with two characters,
- 3-dimensional matrix where each plot is encoded with numerical values.

**Figure 3:**
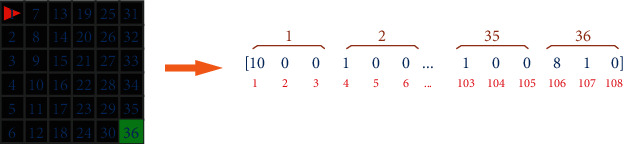
Flattening virtual environment state parameters.

In the application, the state of the virtual environment is obtained as a 3-dimensional matrix. As the number of parameters is much less compared to the RGB image, and the parameters consist of numerical values, it is easy to make them suitable for artificial neural network (ANN) use. As the number of data entered in the ANN increases, the network becomes more complex and the outer wall whose state never changes is removed to make the network simpler. The state parameters of the environment of a 3-dimensional matrix structure are flattened as in Figure 3 so that they can be used in ANNs.

As shown in Figure 3, each plot has three parameters (object, color, direction). These parameters are added consecutively as in the figure, resulting in an array of 108 parameters showing the state of the environment. The array created for the MiniGrid-Empty-16 × 16-v0 environment has 588 parameters.

### 2.2. Actions

Practically, the agent can make 3 different movements. A numerical value is defined for each movement made by the agent. These are

- Turn 90° left ⟶ 0.
- Turn 90° right ⟶ 1.
- Move forward 1 unit ⟶ 2.

### 2.3. Reward Function

In the application, the total reward value that the agent can receive in a level ranges from 0 to 1. In order for the agent to complete the episode, the agent must either reach its target or take the maximum number of steps. The maximum number of steps for the application was determined as 50. If the agent reaches at its target, the reward function is as shown in the following equation:

(1) $$\begin{array}{c} \text{R}=1-0,9*\frac{\text{Number of steps}}{\text{Maximum number of steps}}. \end{array}$$

According to the reward function, as the number of steps increases, the reward earned decreases, thus the agent should find the shortest path to maximize its reward. If the agent takes the maximum number of steps, the episode ends and the agent gets 0 points because it fails.

## 3. DQN and HSP-DQN

**Figure 4:**
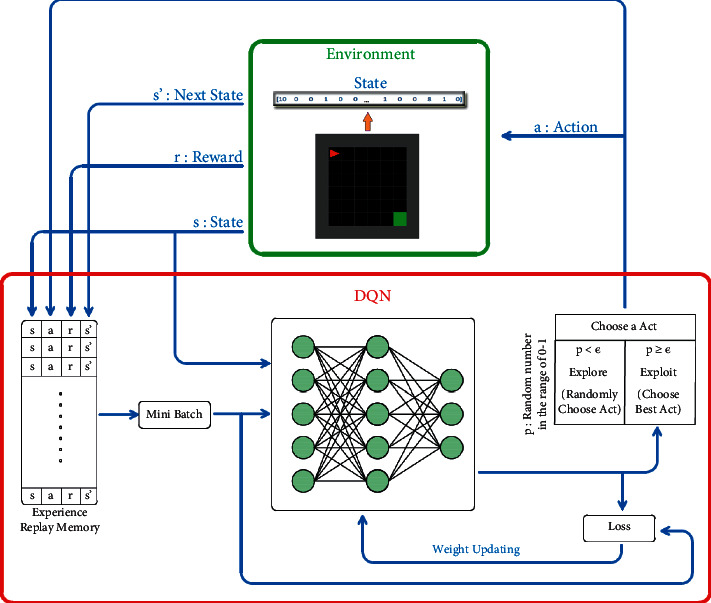
Structure of DQN.

With the advancement in DL, the Markov Decision Process can be solved with deep neural networks [47]. As one of such methods, DQN is successful in some areas at the human level [13]. DQN is a DRL algorithm combining supervised learning and RL techniques [13]. The structure of DQN, which is frequently used in fields such as games [13], robotics [48], and natural language processing [49], is shown in Figure 4.

In the DQN algorithm, the agent makes random movements at the beginning of the training and records its experiences about the environment in the experience replay memory. How much reward (*r*) the action (*a*) brings in a state (*s*) and the new state (*s*′) after the action are recorded in this recording field. When the experiences obtained in this way reach a sufficient number, the experience equal to the size of the mini-batch is randomly selected from the experience replay memory and used for the training of the Q-network. So, the network is not affected by local minimums. Also, in the DQN algorithm, the agent determines its action using Epsilon-Greedy (∈). It ∈ can take values between 0 and 1. ∈ A value close to 1 indicates that the agent focuses on exploration, while a value close to 0 indicates that it focuses on making the best action determined by its experience. For this reason, at the initial stage of training, ∈ the agent is allowed to explore the environment by choosing a value close to 1. As the episodes progress, ∈ its value is proportionally reduced, allowing the agent to choose the best action using its experience. The DQN algorithm is very practical for discrete and small-sized environments due to its described structure.

**Figure 5:**
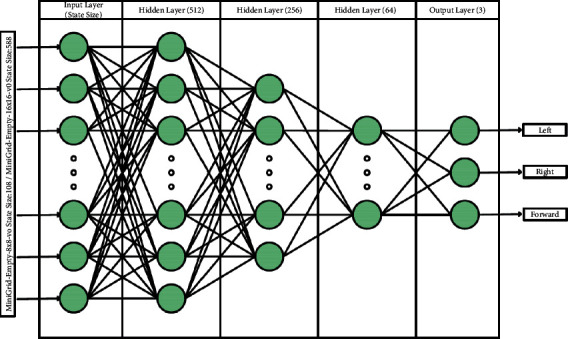
Q-network architecture.

In the application, MiniGrid-Empty-8 × 8-v0 and MiniGrid-Empty-16 × 16-v0 environments are used as virtual environments. The agent was trained separately for the specified environments with the DQN algorithm and the HSP-DQN (Hierarchical Initial Position DQN) algorithm proposed by us, and the algorithm performances were examined. The network architecture in Figure 5 is used for both algorithms.

In the real environment application, it is desired that the mobile robot reaches its target, regardless of its starting position and direction. Therefore, in the training with DQN, the position and the direction of the agent are chosen randomly in each new episode.

**Figure 6:**
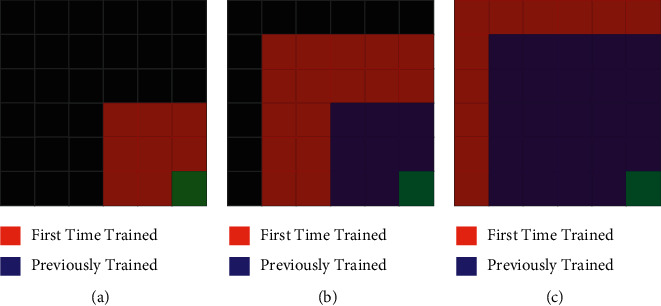
Application of HSP-DQN algorithm to minigrid-empty-8 × 8-v0 environment. (a) Episodes 0–500. (b) Episodes 500–1000. (c) Episodes 1000–3000.

However, in the training with HSP-DQN, a region where the agent has a high probability of reaching the target with random movements is determined, and the training is started in this region. The agent is started in a random position and direction, provided that it stays in the determined region in each new episode. After the network is sufficiently trained for this region, a new training region is determined and ∈  value is increased, allowing for the agent to re-explore. There are two regions at this stage; the region where the training was performed for the first time and the region where the training was already performed. Both regions are used during training. In each new episode, one of the two episodes is chosen alternately and the agent is started in a random position and direction. In this way, the previous information of the network can be kept up to date and new regions can be explored. At this stage, after the network is sufficiently trained, the region trained for the first time is included in the previously trained region. ∈ value is increased again and a new exploration region is determined. Thus, the network is trained gradually. These stages continue until the network learns the whole environment.  Figure 6 shows the application of the HSP-DQN algorithm in the MiniGrid-Empty-8 × 8-v0 environment.

**Figure 7:**
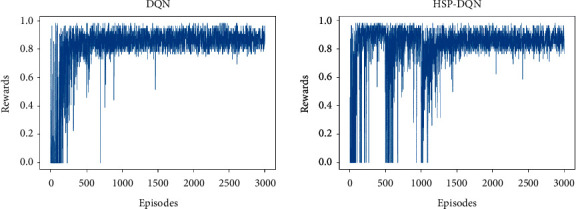
DQN and HSP-DQN reward graphs (minigrid-empty-8 × 8-v0).

Figure 7 shows DQN and HSP-DQN reward graphs for MiniGrid-Empty-8 × 8-v0 environment. As the HSP-DQN algorithm focuses on exploration in each new region definition, sections where it fails or gets low scores are observed. However, the graphics show similarity after 1500 s.

Table 1 shows performance data of DQN and HSP-DQN. In both algorithms, successful results were obtained at the end of 3000 sections. Although the total number of steps of HSP-DQN is slightly less than that of DQN, it does not provide an obvious advantage.

As the MiniGrid-Empty-8 × 8-v0 environment area is not large, the DQN algorithm has been successful in learning the environment.

**Figure 8:**
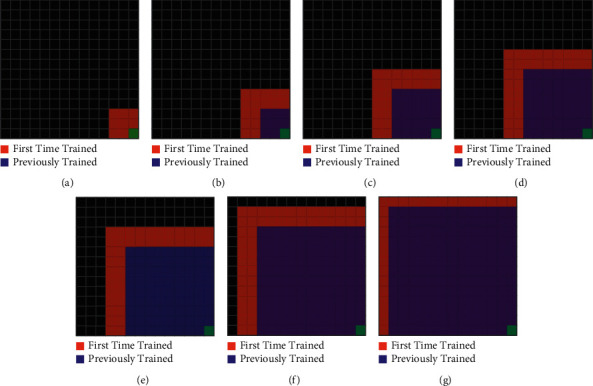
Application of HSP-DQN algorithm in the Minigrid-Empty-16 × 16-v0 environment. (a) Episodes: 0–500. (b) Episodes: 500–1000. (c) Episodes: 1000–2000. (d) Episodes: 2000–3000. (e) Episodes: 3000–4000. (f) Episodes: 4000–5000. (g) Episodes: 5000–8000.

Algorithm performances are also compared for the MiniGrid-Empty-16 × 16-v0 environment with a larger area.  Figure 8 shows the application of the HSP-DQN algorithm in the MiniGrid-Empty-16 × 16-v0 environment.

**Figure 9:**
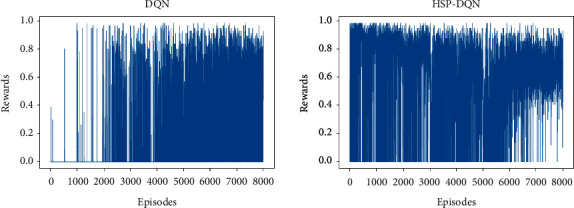
DQN and HSP-DQN reward graphs (Minigrid-Empty-16 × 16-v0).

Figure 9 shows DQN and HSP-DQN reward graphs for MiniGrid-Empty-16 × 16-v0 environment. For the DQN algorithm, the number of successful episodes in the first 2000 episodes is quite low. At the end of 8000 episodes, it is observed that it often fails. In the HSP-DQN algorithm, successful and unsuccessful episodes are observed up to 6000 episodes, but in the following episodes, it is seen that the failures became sparse and the reward interval tended to narrow, that is, the agent explores short paths.

Table 2 shows performance data of DQN and HSP-DQN. Accordingly, the successful episode rate of HSP-DQN is quite high compared to DQN. The total number of steps of DQN at the end of 8000 episodes is approximately 84% more than HSP-DQN. In other words, the training period of the DQN, which has a low success rate, is also quite long compared to the HSP-DQN. Also, at the end of 8000 episodes, the Epsilon-Greedy value decreases to 0.018 in DQN, while this value is 0.178 for HSP-DQN. So, the HSP-DQN is likely to explore shorter paths.

## 4. Robot Design

### 4.1. General Structure of the Robot

Maneuverability is an important feature for mobile robots [50]. Omni-wheeled robots have high maneuverability due to their ability to move directly from one position to another without being redirected [51].

**Figure 10:**
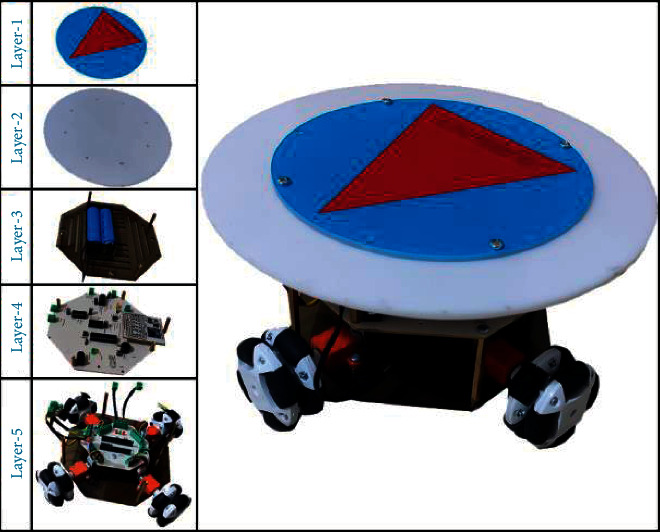
Omni-wheeled mobile robot.

As shown in Figure 10, a mobile robot with high maneuverability was designed to use the agent trained in the virtual environment in the real environment application. The robot consists of five layers. The 1st and 2nd layers are the visual layers and are used for the determination of the robot's position and rotation. Their intended purposes will be explained in the next sections.

The 3rd layer has two serial connected Li-ion batteries with a current capacity of 2500 mAH and a voltage value of 3.7 V, which provide for the energy needs of the robot.

**Figure 11:**
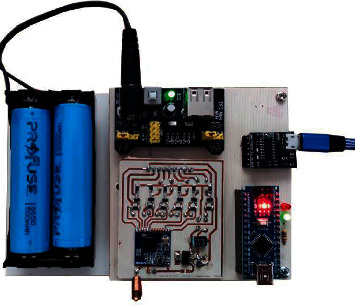
RF controller.

In the 4th layer, there is the robot control circuit that executes commands from the computer via the RF controller, as shown in Figure 11.

RF control circuit and robot control circuit are microcontroller-based circuits. Both circuits use Atmel's ATmega328P microcontroller. The robot control circuit is designed so that it can control DC motors with encoder, control the PWM speed, and give audible and visual warnings. For the communication between the controller and the robot, the SX1278 transceiver module that operates in the 433 MHz frequency band and communicates with Long Range (LoRa) modulation is used.

The 5th layer is the layer containing four DC motors with N20 encoder and four 8-cylinder omni wheels with a 50 mm diameter, which are controlled by these motors.

### 4.2. Movements and Calibration of Mobile Robot

**Figure 12:**
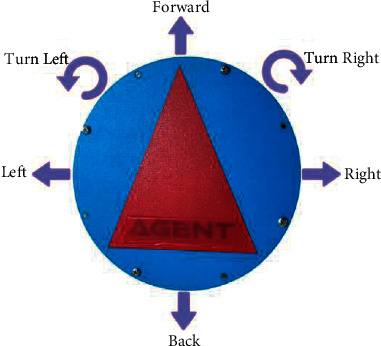
Movements of mobile robot.

In this study, the agent was trained in the empty virtual environment. There are three movements the agent can make for this environment (go forward 1 unit, 90^o^ turn right, 90^o^  turn left). Empty environment is a discrete environment. Nevertheless, because the real environment is a continuous environment, the mobile robot can be in numerous different angular and positional positions. In order for the mobile robot to use the information it receives from the discrete environment, the real environment should be likened to a discrete environment. In other words, the mobile robot should be able to move in a way that can bring to a desired position. The mobile robot was designed to make 6 different movements, as shown in Figure 12, to go to the desired location. It can make turns with an accuracy of approximately 3° and linear movements with an accuracy of about 1 cm in the range of 0°–360°.

**Figure 13:**
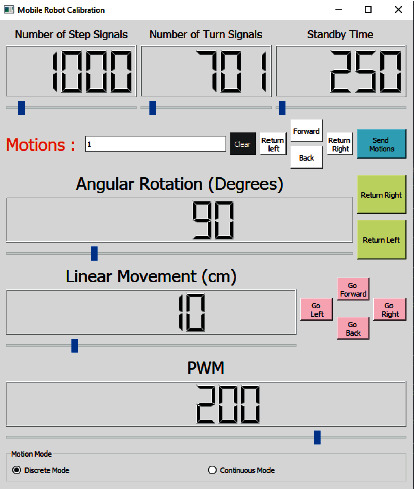
Mobile robot calibration program.

Although omni-wheeled robots have high maneuverability, the floor under them can negatively affect their movements [52]. Although the designed mobile robot has motors with encoder, encoder signals cannot guarantee the position of the robot. Due to the floor under the robot and the standing positions of the omni wheels, undesirable situations such as idle rotation of the wheels during take-off may be encountered. To reduce the mentioned problems relatively, the mobile robot calibration program shown in Figure 13 was prepared. By using this program, the number of signals from the encoder for 1 unit step of the robot (determined as 30 cm for this study), and the number of signals from the encoder for 90^*o*^ rotation can be found. Multiple movements can be sent and it can be waited for the specified time or not waited in transition between movements depending on the selected mode (discrete, continuous). Angular and linear movements at different values can be determined and the speed of motors can be controlled with PWM.

**Figure 14:**
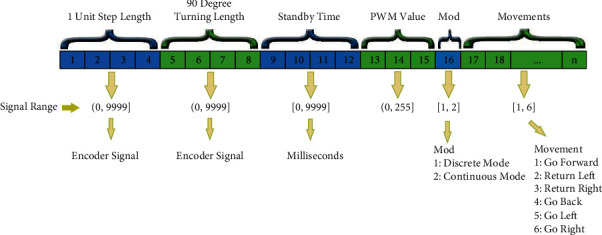
RF data packet.

To control the abovementioned features of the mobile robot, a data package is designed as in Figure 14. This data packet is prepared according to the action to be taken by the robot and sent with RF signals, and the robot can perform the necessary actions by decoding these signals.

Table 3 shows the experimentally obtained encoder signal values to be used in the study.

**Figure 15:**
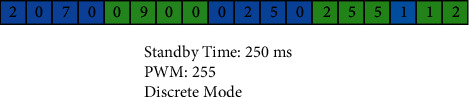
Sample data package.

For example, the data packet required for the mobile robot to move 1 unit forward and turn 90° left is shown in Figure 15.

## 5. Real Environment Application with HSP-DQN

This section shows that the HSP-DQN network trained in a discrete empty environment can also be used in a real environment. Here, the HSP-DQN network was trained in a discrete environment and its application was performed by likening the real environment of the application to a discrete structure.

**Figure 16:**
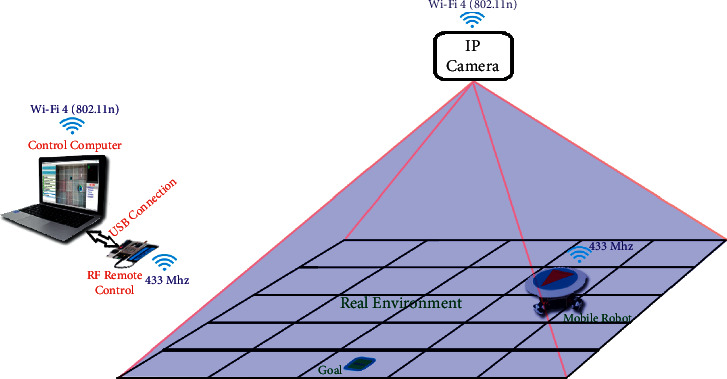
Real environment structure.

Figure 16 shows the real environment, which will be likened to a discrete structure. The environment data are obtained by the IP camera that takes the top view of the environment. However, the mobile robot used in the real environment receives the action commands from the computer via RF signals.

Simulation of real environment to discrete structure:

- Detection of mobile robot and target with convolutional neural networks,
- Plotting the real environment as in the virtual environment,
- Correction of the position and angle of the mobile robot to fit in the discrete environment,
- The real environment is matched with the virtual environment in stages.

At the end of these stages, the mobile robot is able to use the HSP-DQN network trained in the virtual environment. Explanations about the stages are given in this section.

### 5.1. Detection of Objects with Convolutional Neural Networks

Convolutional Neural Networks (CNN) form the basis of image classification by DL [53]. The effectiveness of CNNs was proven in the ImageNet Large Scale Visual Recognition Challenge (ILSVRC) competition held in 2012 [54]. After this success, studies with CNN gained acceleration [55–59].

Today, CNN models developed for object detection have reached an enormous position due to their success in classification. However, model sizes are also increasing. This limits its use in real-world applications where latency and resource usage are important, such as autonomous robots. Therefore, model efficiency is becoming increasingly important in real-life applications [60].

In our study, EfficientDet D0 CNN model was used for object detection. This model, which uses the EffiCientNet model as the backbone network, has a good performance in image classification by scaling the network width, depth, and input resolution together. As the number of parameters of the model is optimized, it is also advantageous in terms of using system resources [60–62].

**Figure 17:**
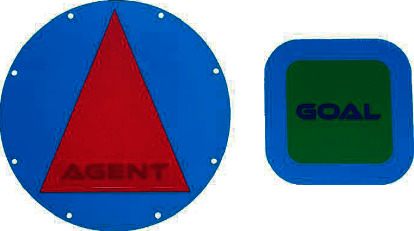
Agent and target objects.

The CNN model was trained to detect the agent and target objects shown in Figure 17. 117 and 32 images were used for training and testing, respectively, in different resolutions and light conditions.

**Figure 18:**
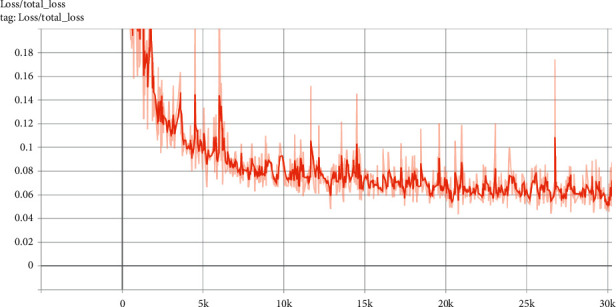
Total-loss graph.

The total-loss graph of the model trained on 30,000 epochs is shown in Figure 18. The graphs show that the model has been trained to a large extent after 6,000 epochs.

Using the CNN model, the presence of the Agent and target in the environment, the position and the center information of the objects in the image are determined. In addition, the pixel distance of the environment can be converted by taking the agent image as a reference.

### 5.2. Plotting of Real Environment

**Figure 19:**
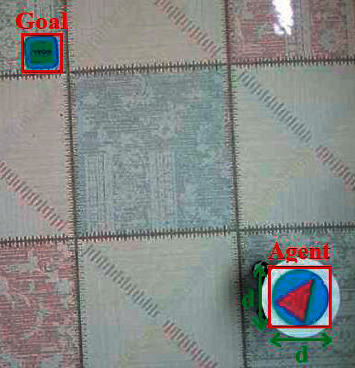
Detection of agent and target with CNN.

The environment where the HSP-DQN network is trained is a discrete environment. However, in order for the mobile robot to use the trained HSP-DQN network, the real environment should be likened to the discrete environment. The steps of the simulation process are described below;

Stage-1: Real environment data are obtained using only the top view of the environment. Using the CNN from this image, the positions of the agent and the target are determined as in Figure 19.
Stage-2: Centers of the target (*Goal*_*x*_, *Goal*_*y*_) and the agent (Agent_*x*_, Agent_*y*_) are calculated for use in the next stages. The area of each plot in the real environment is determined as 30 × 30 cm^2^. In other words, the mobile robot should travel 30 cm to pass from the center of one plot to the center of the other plot. The step distance set as 30 cm (Step_*d*_) is calculated by taking the agent image as a reference. The diameter of the circular agent image is 16 cm and the length of one side (*d*) of the frame that detects the agent is approximately equal to the diameter of the agent image. So 1 cm is approximately equal to *d*/16 pixels. The step distance of the mobile robot in pixels is shown in the following equation:
  (2) $$\begin{array}{c} {\text{Step}}_{d}=30\text{ cm }⟶\;{\text{Step}}_{d}=\frac{30}{16}*\text{dpixel}. \end{array}$$
Stage-3: The centers of the plots in the real environment including width (Image_*w*_) and length (Image_*h*_) of the environment image are determined using equation (3). As the location of the agent is fixed when the real environment is plotted, a list of the plot centers (*G*_*centers*_) is created by taking the center of the target as a reference, and the real environment is plotted.
  (3) $$\begin{array}{c} G_{centers}=\left\{\begin{array}{c} a,b|a,b\;\in \text{ Ν,}\;\left(0<{\text{Goal}}_{x}\pm {\mathrm{a*Step}}_{d}<{\text{Image}}_{w},0<{\text{Goal}}_{y}\pm {\mathrm{b*Step}}_{d}<{\text{Image}}_{h}\right). \end{array}\right\} \end{array}$$
Stage-4: The location of the agent may not match the center of any plot because the center of the target is used as the reference in the plotting process. For this reason, the mobile robot should be positioned according to the nearest plot. Using equation (4), the distances of the mobile robot to the plot centers ((Center_*d*_)) are listed.
  (4) $$\begin{array}{c} {\text{Center}}_{d}=\left\{\begin{array}{c} \left(k,l\right)\in |\;G_{centers}\;\sqrt{{\left({\text{Agent}}_{x}-x_{k}\right)}^{2}-{\left({\text{Agent}}_{y}-y_{l}\right)}^{2},} \end{array}\right\} \end{array}$$

The element of the *G*_*centers*_ list Center_*d*_ as the minimum value in the list shows the plot center closest to the mobile robot. This plot also shows the initial position of the mobile robot.

**Figure 20:**
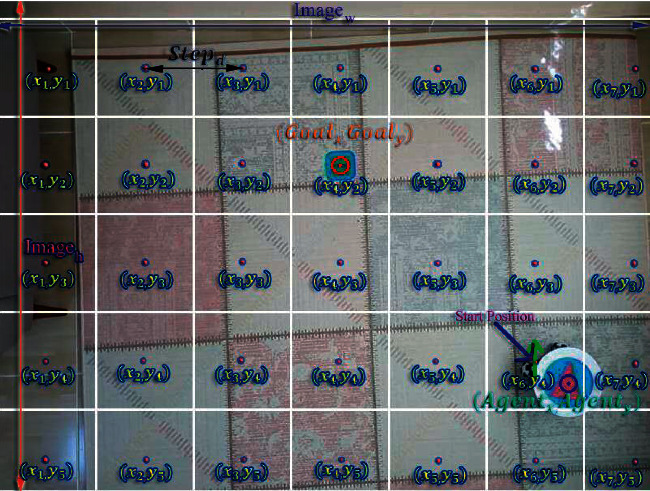
Plotting of the real environment.

The plotted real environment is shown in Figure 20. After the real environment is plotted, the location where the center of the robot should be at the beginning is determined.

### 5.3. Determination of the Rotation of the Mobile Robot

CNNs have become the main method in the field of image recognition thanks to their strong feature extraction ability. Most CNNs do not deal with the angle of the image and just focus on image recognition. However, angle data are also crucial in robot applications [63].

In the present study, the agent was trained with HSP-DQN in a discrete virtual environment. Only four directions exist for the virtual environment. However, in the real environment, the mobile robot can take an unlimited angular position. In order for the mobile robot to use the information in the virtual environment, it should adjust its direction to resemble the most suitable direction in the virtual environment. For this reason, a structure that can detect the angle of the mobile robot is required. The image used by CNN for the detection of the mobile robot is also used in the algorithm created for the detection of the mobile robot's angle. The angle and direction of the isosceles triangle on the mobile robot are also the same as the angle and direction of the mobile robot. The purpose of the algorithm is to determine the direction and angle of the mobile robot by determining the direction and angle of the triangle in the image.

**Figure 21:**
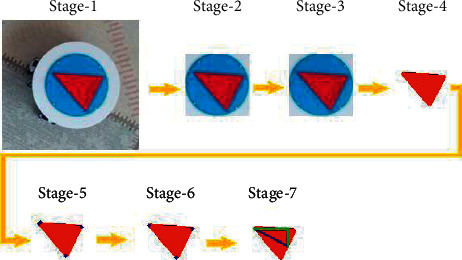
Stages of angle determination algorithm.

Figure 21 shows the stages of the algorithm.

  Stage-1: A top image of the mobile robot and the environment where the target is located is taken.
  Stage-2: The coordinates of the agent are determined by CNN and this part is taken from the image. Just below the agent image, a white layer is used, which visually covers the part under the robot. Thus, while the algorithm is running, it is not affected by the colors on the ground and the mobile robot can use the algorithm on any color ground.
  Stage-3: Bilateral Filter is applied to reduce noise and sharpen edges in the image [64].
  Stage-4: As the mobile robot image is in the shape of a circle, the frame determined by CNN is roughly like a square, the center of the frame always remains inside the triangle shape. *R*, *G*, and *B* values are listed separately by retrieving the RGB values of the pixel in the center of the image and its eight neighbors. The values in each list are ordered in ascending order. The reference RGB values of the triangle image are obtained by retrieving the 5th elements of the lists created for *R*, *G*, and *B*. This process aims to establish a structure that can work in different light environments. A two-color image is obtained by assigning red color value to pixels that are close to the reference RGB values and white color value to other pixels.
  Stage-5: The pixels where the red color is first detected are determined by scanning the image separately from the right, left, bottom, and top. Thus, four points are obtained.
  Stage-6: The closest two points within the four detected points show the same corner. Only one of the two points can be used. To make a selection, the distances of two points to other points are calculated separately and the point that is farther from other points is selected as the 3^rd^ corner point because being further away implies that the point is further away.
  Stage-7: The distances between the corner points are calculated. As the twin sides of the triangle are longer than the base side, the side with the two closest vertices shows the base of the triangle and the other vertex shows the vertex of the triangle. By drawing a line to the midpoint of the vertex and the base, a Pythagorean triangle is formed as in Figure 21 Stage-7, and the angle of the triangle, hence the angle of the mobile robot, is determined by Arctangent.

**Figure 22:**
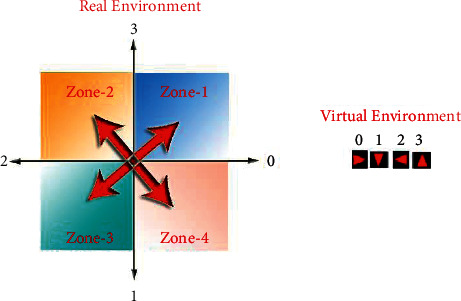
Directions in real environment vs. virtual environment.

If the angle found is 0°, the position of the mobile robot in real environment and virtual environments can be matched. If the angle found is different from 0°, the direction of the mobile robot can be in one of the four regions as shown in Figure 22. The triangle direction determines which region the mobile robot is in. The direction to which the mobile robot will turn is determined by comparing which direction it is angularly close to.

Using the algorithm, it is determined in which region the mobile robot is, how much the robot should turn angularly to the right or left to match the most suitable direction in the virtual environment, and in which position the mobile robot will be after the rotation is corrected.

**Figure 23:**
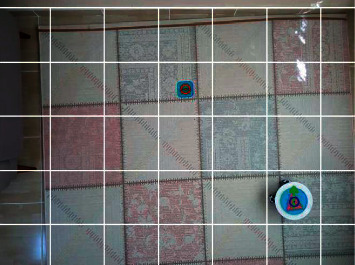
Positioning the mobile robot to the initial position.

The mobile robot autonomously adjusts its angle using the values determined in this episode and then positions it to the initial position determined in the previous episode with linear movements (right, left, forward, backward). After the mobile robot corrects its angle and position, both the mobile robot and the target are located in the center of the plot they are in, as shown in Figure 23. The mobile robot is also positioned in one of the four directions it can take, as in the virtual environment.

**Figure 24:**
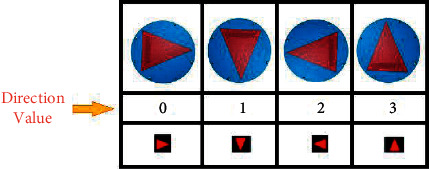
Directions for mobile robot and virtual agent.

As the mobile robot and the target are located only in the centers of the plots and there are four directions that the mobile robot can take, as shown in Figure 24, the real environment and the discrete virtual environment can be matched.

### 5.4. Matching the Real Environment with the Virtual Environment and Determining the Path Plan

**Figure 25:**
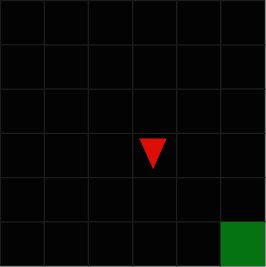
Virtual environment.

To use the HSP-DQN network trained in the virtual environment in the real environment, it is necessary to match the position of the mobile robot with the position of the virtual agent, and the position of the real target with the position of the virtual target. For the virtual environment, the agent can be in any position and direction at the beginning, and the target is located in the lower right corner of the virtual environment as in Figure 25.

**Figure 26:**
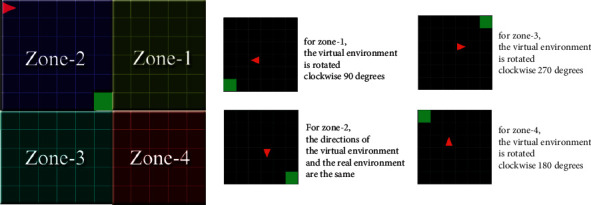
Rotation of the virtual environment to obtain regions in the real environment.

However, in the real environment, the mobile robot can be in any direction and position around the target. In other words, many situations exist where the real and virtual environments cannot match. To solve this problem, the real environment is divided into four regions, as in Figure 26, according to the position of the mobile robot to the target. The virtual environment is rotated so that it can match the real environment.

**Figure 27:**
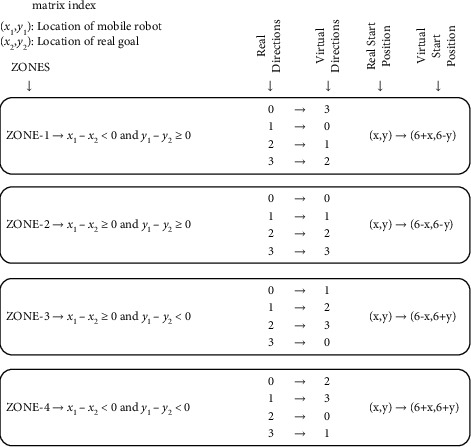
Conversion of virtual environment according to real environment.

To match the rotated virtual environment with the real environment, the initial direction and the position of the virtual agent are converted according to the region as in Figure 27.

**Figure 28:**
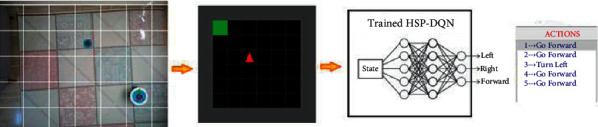
Use of HSP-DQN in real environment.

As a result of these conversions, all states in the real environment can be matched with the virtual environment and the trained HSP-DQN network can be used for the real environment, as in Figure 28.

The path plan is determined in the virtual environment before the mobile robot takes action. With the trained HSP-DQN, an action is generated for the current state of the agent and applied in the virtual environment, enabling the agent to move to its new position. The HSP-DQN continues to generate action until the agent reaches its target, and these actions are listed for the mobile robot to use. In this way, the path plan of the mobile robot is obtained.

After the path plan is drawn, the actions to be taken are sent to the mobile robot in order. After each action, the angle and the position of the mobile robot are checked and deviations are corrected. Whether the mobile robot completes its current action or not is determined by the background difference method. The mobile robot is ensured to reach the target by sending the next action it needs to do after each completed action.

## 6. Discussion and Future Work

In the previous sections of the paper, the proposed algorithms have been discussed. In this section, the performance of the system will be examined. Although DQN algorithm is successful in MiniGrid-Empty-8x8-v0 environment, it cannot achieve similar success in MiniGrid-Empty-16 × 16-v0 environment because the agent can only receive rewards when it arrives at the destination in MiniGrid-Empty environments. If the environment has a large area such as MiniGrid-Empty-16 × 16-v0 and the rewards are sparse, it is difficult for the DQN algorithm to generate enough meaningful data to train the network with random movements, and the convergence speed becomes very slow. In the proposed method, the initial position of the agent is started in the region where the probability of reaching the reward is high and this increases the probability of the network to reach meaningful data. Then, as the network learns the paths in the environment the area where the agent can start is gradually expanded. Thus, for large areas with sparse rewards the convergence problem of the network is addressed. Also, performance analyses are performed for MiniGrid-Empty-16 × 16, which is a larger environment than MiniGrid-Empty-8x8.

In the experimental study, a computer with Windows 10 Pro 64 Bit operating system was used. The hardware specifications are provided in Table 4.

Neural networks used in DQN and HSP-DQN algorithms have the same features. The information about the structure of the neural network is shown in Table 5 and the hyper parameters of the algorithms are shown in Table 6.

In the DQN algorithm, the number of different states that the agent can start is 780. While the neural network is being trained, one of the 780 states is randomly selected as the initial state in each new section. In the proposed algorithm, as the sections progress, the area where the training can begin is gradually enlarged. Thus, the number of different states that the agent can initiate increases, as shown in Table 7.

In both algorithms, the performances of the models were examined after training 8000 sections under the abovementioned conditions. The time spent by the algorithms for training the neural network is shown in Table 8. The training of the proposed algorithm was completed in 45.78% less time than the DQN algorithm. Although the agent moved more in the environment in the DQN algorithm, the time spent in the sections increased as it reached fewer targets compared to the proposed algorithm.

**Figure 29:**
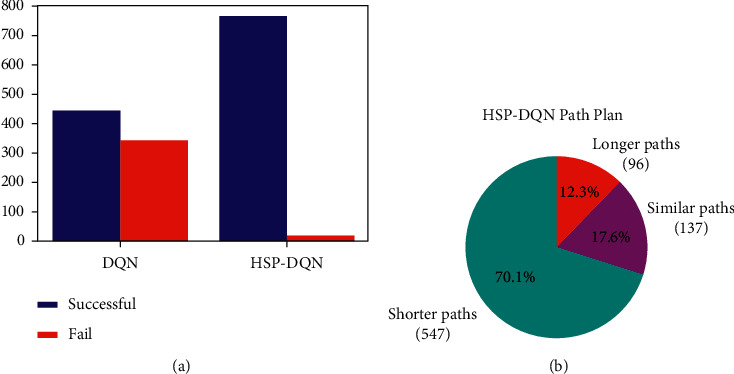
Virtual environment (minigrid-empty-16 × 16-v0) performances of HSP-DQN, and DQN.

In order to compare the success of the trained models, all conditions were tested in the environment with 780 different initial conditions. The related results are presented in Table 9. From the table it can be seen that success rate of the proposed algorithm is 41.28% higher than the DQN algorithm. Success of the algorithms is also graphically shown in Figure 29(a).

Moreover, the lengths of the paths detected by the algorithms were also examined. The models trained with DQN and the proposed algorithm were started with the same initial conditions and the obtained path lengths were compared as shown in Figure 29(b). The proposed algorithm finds a shorter path compared to the DQN algorithm in 70.1% of different initial conditions.

As a result of the tests, it was seen that the success rate and convergence speed of the proposed algorithm are better than the DQN algorithm for large environments with sparse rewards. In addition, the effects of some DRL-based algorithms developed with our study on success rates are shown in Table 10.

**Figure 30:**
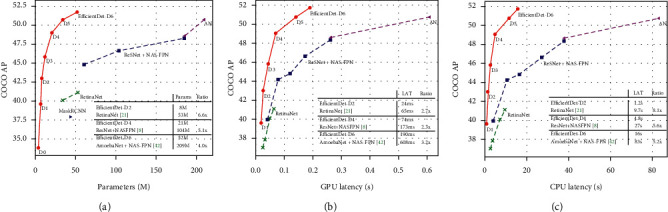
Performance of the efficientdet model on the COCO dataset [60]. (a) Model size. (b) GPU latency. (c) CPU latency.

In the real environment application of the study, only the top view of the environment was used as the system input. Since it was desired that the application run in real time, this image should be used efficiently. The object detection capability, speed, and model size of the model used for object detection were very important. Therefore, the EfficientDet-D0 model was used for object detection by considering these criteria. The performance analyses of the EfficientDet model presented in the original article are shown in Figure 30. The model is considered to be suitable for mobile robot applications with limited resources in terms of model size, speed, and object detection.

Performance of the EfficientDet model in our application was also tested. The model was trained as specified in Section 5.1. The trained model detected objects with the image taken from the IP camera mounted at a height of 180 cm from the ground. The captured image had a resolution of 800*∗*600 pixels and covered an area of 2.1 × 1.6 m^2^. In the performed tests, the model could successfully detect mobile robot and target objects, provided that the ambient lighting is appropriate. Also, it was determined that the object recognition process was completed in the time interval of 820–920 ms.

As mentioned earlier, there are 780 different initial conditions in the MiniGrid-Empty-16 × 16 virtual environment. With the employment of the structure described in Section 5.4, the target is ensured to be in any position in the environment, rather than just in a certain position. In other words, the target can be positioned in 196 different positions at the beginning. Thus, the success of the trained network is ensured for 780 × 196 = 152.880 different initial conditions. In here, the recommended regional matching process takes less than 1 ms to complete.

The real environment processes are performed in different steps.  Table 11 provides the processing times for those steps. The designed system is capable of detecting the state of the environment within 1–1.5 seconds in order to allow the robot to perform a specific task.

A central computer is used for the designed system in order to perform the semantic data extraction, determination of the mobile robot motion, and notification of the determined motion. The mobile robot acts as an actuator by using the commands from the central computer. As the mobile robot does not require any computational load, a low-cost robot was designed with the part listed in Table 12.

LoRa-based modulation is used for the wireless communication between the computer and the mobile robot. The LoRa has advantages such as wide coverage, low power consumption, low cost, and no license requirement [72]. However, it has a data transfer rate of 290 kbps which is slower than many wireless communication methods [73]. Despite the latter disadvantage, LoRa still is an ideal communication method for the application reported in this work since only small-sized data signals are transmitted for the movement of the robot.

In the real environment experiments, the DRL-based mobile robot was launched with different initial conditions for the four regions that are specified in Section 5.4. As can be observed from the following link, the mobile robot was able to successfully reach the target in all experiments (Please see: https://youtu.be/bEAmJF6lD4g). The following were also observed in the experiments:

- The omni-wheeled structure of the mobile robot has advantages in terms of maneuvering. However, it is prone to disturbances from the floor on which the robot is operating. Although this situation does not prevent the robot from reaching its target, it negatively affects the time of the robot to reach the target.
- Thanks to the adaptive structure of the system, the working area of the system can be changed by changing the height of the camera from the ground. However, excessive magnification of the area covered by the camera or improper lighting of the environment may adversely affect the detection of objects in the environment.

This work forms a good basis for the future works as it demonstrates the possibility of controlling many robots within a long range by using a single center. The designed robot has the potential to be used in swarm robotics and multi-agent systems, as it is cost-effective and long-range controllable; thus, this work presents a good contribution to one of the important research topics known as multi-robot applications [74–76]. The latter is important for accelerating global policy education, as the use of large numbers of robots can provide greater data diversity [77]. In the future, real-world applications of DRL algorithms, which will include sequential tasks to enable the agent to reach its target optimally in environments with static and dynamic obstacles or control more than one mobile robot, can be performed as a future work.

## 7. Conclusion

In this study, the HSP-DQN algorithm, which had a higher convergence rate and success rate than the classical DQN algorithm, was proposed for the environments with sparse rewards. With the proposed algorithm, a real environment application was performed for the first time by using the network parameters of an agent trained in the minimalistic grid world virtual environment. A low-cost mobile robot that can be controlled within a long range by a central computer was designed for this purpose. Also, a system which can efficiently match the virtual environment with a discrete structure and the real environment was designed.

## Figures and Tables

**Table 1:** Comparison of DQN and HSP-DQN (MiniGrid-Empty-8 × 8-v0).

Minigrid-Empty-8 × 8-v0	DQN	Episodes	1–500	500–1000	1000–2000	2000–3000
		Epsilon-greedy	1 > *ε* > 0.779	0.779 > *ε* > 0.606	0.606 > *ε* > 0.368	0.368 > *ε* > 0.223
		Total number of steps	10646	4127	7373	7305
		Number of successful episodes	387	499	1000	1000
		Success rate (%)	77.40	99.80	100	100
	Total number of steps				29451	
	Average number of steps per episode				9.82	
	HSP-DQN	Episodes	1–500	500–1000	1000–2000	2000–3000
		Epsilon-greedy	1 > *ε* > 0.779	0.800 > *ε* > 0.623	0.800 > *ε* > 0.485	0.485 > *ε* > 0.294
		Total number of steps	6083	5037	9847	7659
		Number of successful episodes	441	483	993	1000
		Success rate (%)	88.20	96.60	99.30	100
	Total number of steps				28626	
	Average number of steps per episode				9.54	

**Table 2:** Comparison of DQN and HSP-DQN (MiniGrid-Empty-16 × 16-v0).

MiniGrid-Empty-16 × 16-v0	DQN	Episodes	1–500	500–1000	1000–2000	2000–3000	3000–4000	4000–5000	5000–6000	6000–7000	7000–8000
		Epsilon-greedy	1 > *ε* > 0.779	0.779 > *ε* > 0.606	0.606 > *ε* > 0.368	0.368 > *ε* > 0.223	0.223 > *ε* > 0.135	0.135 > *ε* > 0.082	0.082 > *ε* > 0.050	0.050 > *ε* > 0.030	0.030 > *ε* > 0.018
		Total number of steps	24973	24912	49430	46311	45486	42255	37297	32136	28881
		Number of successful episodes	2	2	16	119	144	261	424	569	708
		Success rate (%)	0.40	0.40	1.60	11.90	14.40	26.10	42.40	56.90	70.80
	Total number of steps				331681						
	Average number of steps per episode				41.46						
	HSP-DQN	Episodes	1–500	500–1000	1000–2000	2000–3000	3000–4000	4000–5000	5000–6000	6000–7000	7000–8000
		Epsilon-greedy	1 > *ε* > 0.779	0.800 > *ε* > 0.623	0,800 > *ε* > 0,485	0,800 > *ε* > 0,485	0,800 > *ε* > 0,485	0,800 > *ε* > 0,485	0,800 > *ε* > 0,485	0.485 > *ε* > 0.294	0.294 > *ε* > 0.178
		Total number of steps	8806	7157	17491	19176	26923	26424	34068	20562	19607
		Number of successful episodes	388	442	835	842	709	766	577	970	994
		Success rate (%)	77.60	88.40	83.50	84.20	70.90	76.60	57.70	97.00	99.40
	Total number of steps				180214						
	Average number of steps per episode				22.53						

**Table 3:** Experimentally obtained encoder signal values.

Action	Encoder signal
1 cm linear motion	22
30 cm linear motion (one unit)	2070
3° turning motion	10
90° turning movement	900

**Table 4:** Specifications of the computer used in the experimental study.

Hardware	Specifications
CPU	Intel™ Core™ i7-9750H CPU @ 2.60 GHz 2.60 GHz
GPU	NVIDIA GeForce RTX 2070 8 GB
RAM	16 GB 2667 MHz

**Table 5:** Features of the neural network used in DQN and HSP-DQN.

Layers	Number of neurons	Activation function	Parameters
Input layer	512	ReLU	301568
Hidden Layer-1	256	ReLU	131328
Hidden Layer-2	64	ReLU	16448
Output layer	3	Linear	195
Total parameters			449.539
Optimizer: RMSprop algorithm			
Loss: Mean squared error			
Learning rate: 0.00025			
Rho: 0.95			
Epsilon: 0.01			

**Table 6:** Hyper parameters of DQN and HSP-DQN algorithms.

Hyper parameters	Value
Total episodes	8000
Max step	50
Memory	2000
Epsilon greedy	1.0
Minimum epsilon greedy	0,001
Epsilon greedy decay	0,9995
Mini batch	64

**Table 7:** Gradual enlargement of the training area.

Education levels	Episodes	Area	Number of different states the agent can start
1^st^ level	1–500	3 × 3	32
2^nd^ level	501–1000	5 × 5	96
3^rd^ level	1001–2000	7 × 7	192
4^th^ level	2001–3000	9 × 9	320
5^th^ level	3001–4000	11 × 11	480
6^th^ level	4001–5000	13 × 13	672
7^th^ level	5001–8000	14 × 14	780

**Table 8:** Training times of DQN and HSP-DQN.

Algorithm	Episodes	Total step	Training time (hours)
DQN	8000	331681	8,3 hours
HSP-DQN	8000	180214	4,5 hours

**Table 9:** Success rates of DQN and HSP-DQN algorithms.

Algorithm	Succeeded	Failed	Success rate (%)
DQN	442	338	56,67
HSP-DQN	764	16	97,95

**Table 10:** The effect of improved DRL-based algorithms on the success rate.

Reference	Authors	Compared algorithm (success rate %)	Proposed algorithm (success rate %)	Simulation	Real system
[65]	Pfeiffer et al.	IL or CPO (37)	IL + CPO (90)	Y	Y
[66]	Hsu et al.	A3C (17.5)	LSTM + DRL (49.5)	Y	Y
[67]	Long et al.	NH-ORCA (78)	Parallel PPO (96.5)	Y	N
[68]	Wang et al.	DDPG (42.67)	Fast-RDPG (97.42)	Y	N
[69]	Lin et al.	PPO (23)	Improved PPO (88)	Y	Y
[70]	Wang et al.	POfD (58.5)	LwH (96.5)	Y	N
[71]	Leiva and Ruiz-del-Solar	DDPG (69)	PCL-LSTM (88)	Y	Y
[29]	Wang et al.	DQN (63.4)	DDQN with PER (81.1)	Y	N
[32]	Kim et al.	DQN (46)	DQN + GRU + action skipping (87)	Y	N
[30]	Zhang et al.	DQN (75)	Improved DQN (90)	Y	N
[7]	Zhou et al.	DDPG (84.25)	LDDPG + D (94.25)	Y	N
[5]	Gong et al.	DDPG (83.5)	MN-LSTM-DDPG (90.5)	Y	N
[26]	Xing et al.	DQN (75)	Area division DQN (100)	Y	Y
Our study	Erkan and Arserim	DQN (56.67)	HSP-DQN (97.95)	Y	Y

**Table 11:** Time of each operation.

Operation	Min. tim (ms)	Max. time (ms)
Object detection	820	920
Parceling the environment	15	16
Ro determining the angle of the robot	40	60
Area determination	0	1
Path planning	100	300
Sending the motion signal from the computer to the controller and from the controller to the robot	100	150
Decoding incoming motion signal by robot	10	20
	1085 ms < total time < 1467 ms	

**Table 12:** Cost of the mobile robot.

Parts	Cost ($)
Robot body + omni wheels (4 pieces)	20
N20 micro encoder gear motor 6V 105 rpm (4 pieces)	24
Electronic components	28
Total	72

## Data Availability

The data used to support the findings of this study are available from the corresponding author upon request.

## References

[1] Zheng J., Mao S., Wu Z., Kong P., Qiang H. (2022). Improved path planning for indoor patrol robot based on deep reinforcement learning. *Symmetry*.

[2] Maddikunta P. K. R., Pham Q. V., B P. (2022). Industry 5.0: a survey on enabling technologies and potential applications. *Journal of Industrial Information Integration*.

[3] Zehra W., Javed A. R., Jalil Z., Khan H. U., Gadekallu T. R. (2021). Cross corpus multi-lingual speech emotion recognition using ensemble learning. *Complex Intell. Syst.*.

[4] Zhu K., Zhang T. (2021). Deep reinforcement learning based mobile robot navigation: a review. *Tsinghua Science and Technology*.

[5] Gong H., Wang P., Ni C., Cheng N. (2022). Efficient path planning for mobile robot based on deep deterministic policy gradient. *Sensors*.

[6] Zhang L., Zhang Y., Li Y. (2020). Path planning for indoor Mobile robot based on deep learning. *Optik*.

[7] Zhou Q., Lyu L., Liu H. Deep reinforcement learning with long-time memory capability for robot mapless navigation.

[8] Sutton A. G., Richard S., Barto A. G. (1998). *Reinforcement Learning: An Introduction*.

[9] Duan Y., Chen X., Houthooft R., Schulman J., Abbeel P. Benchmarking deep reinforcement learning for continuous control.

[10] Lillicrap T. P., Jonathan J, Alexander P. Continuous control with deep reinforcement learning.

[11] Li H., Cai R., Liu N., Lin X., Wang Y. (2018). Deep reinforcement learning: algorithm, applications, and ultra-low-power implementation. *Nano Communication Networks*.

[12] Heuillet A., Couthouis F., Díaz-Rodríguez N. (2021). Explainability in deep reinforcement learning. *Knowledge-Based Systems*.

[13] Mnih V., Kavukcuoglu K., Silver D. (2015). Human-level control through deep reinforcement learning. *Nature*.

[14] Mnih V., Koray K., David S. (2013). Playing Atari with Deep Reinforcement Learning. https://arxiv.org/abs/1312.5602.

[15] Silver D., Thomas H., Julian S. (2017). Mastering Chess and Shogi by Self-Play with a General Reinforcement Learning Algorithm.

[16] Silver D., Schrittwieser J., Simonyan K. (2017). Mastering the game of Go without human knowledge. *Nature*.

[17] Pan X., You Y., Wang Z., Lu C. Virtual to real reinforcement learning for autonomous driving.

[18] Qureshi A. H., Nakamura Y., Yoshikawa Y., Ishiguro H. (2018). Intrinsically motivated reinforcement learning for human–robot interaction in the real-world. *Neural Networks*.

[19] Guo T., Jiang N., Li B., Zhu X., Wang Y., Du W. (2021). UAV navigation in high dynamic environments: a deep reinforcement learning approach. *Chinese Journal of Aeronautics*.

[20] Woo J., Yu C., Kim N. (2019). Deep reinforcement learning-based controller for path following of an unmanned surface vehicle. *Ocean Engineering*.

[21] Wang J., Elfwing S., Uchibe E. (2021). Modular deep reinforcement learning from reward and punishment for robot navigation. *Neural Networks*.

[22] Srinivasu P. N., Bhoi A. K., Jhaveri R. H., Reddy G. T., Bilal M. (2021). Probabilistic Deep Q Network for real-time path planning in censorious robotic procedures using force sensors. *Journal of Real-Time Image Processing*.

[23] Li P., Ding X., Sun H., Zhao S., Cajo R. (2021). Research on dynamic path planning of mobile robot based on improved DDPG algorithm. *Mobile Information Systems*.

[24] Jesus J. C., Bottega J. A., Cuadros M. A. S. L., Gamarra D. F. T. Deep deterministic policy gradient for navigation of mobile robots in simulated environments.

[25] Wang W., Wu Z., Luo H., Zhang B. (2022). Path planning method of mobile robot using improved deep reinforcement learning. *Journal of Electrical and Computer Engineering*.

[26] Xing Y., Young R., Nguyen G. (2022). Optimal path planning for wireless power transfer robot using area division deep reinforcement learning. *Wireless Power Transfer*.

[27] Huang R., Qin C., Li J. L., Lan X. (2021). Path planning of mobile robot in unknown dynamic continuous environment using reward-modified deep Q-network. *Optimal Control Applications and Methods*.

[28] Yu J., Su Y., Liao Y. (2020). The path planning of mobile robot by neural networks and hierarchical reinforcement learning. *Frontiers in Neurorobotics*.

[29] Wang Y., Fang Y., Lou P., Yan J., Liu N. (Jun. 2020). Deep reinforcement learning based path planning for mobile robot in unknown environment. *Journal of Physics: Conference Series*.

[30] Zhang H., Wang P., Ni C., Cheng Hongbo Zhang N., Cheng N. (2022). Deep Q network algorithm based on sample screening. *International Conference on Computer Application and Information Security*.

[31] Ruan X., Lin C., Huang J., Li Y. Obstacle avoidance navigation method for robot based on deep reinforcement learning.

[32] Kim I., Nengroo S. H., Har D. Reinforcement learning for navigation of mobile robot with LiDAR.

[33] Brockman G. (2016). *OpenAI Gym*.

[34] Chevalier-Boisvert M., Dzmitry B., Salem L. (2019). *BabyAI: A platform to study the sample efficiency of grounded language learning*.

[35] Co-Reyes J. D., Abhishek G., Suvansh S. (2019). Guiding policies with language via meta-learning. *ICLR*.

[36] Mul M., Bouchacourt D., Research F. A. I., Bruni E. (2019). Mastering Emergent Language: Learning to Guide in Simulated Navigation. https://arxiv.org/abs/1908.05135.

[37] Hutsebaut-Buysse M., Mets K., Latré S. (2019). Fast Task-Adaptation for Tasks Labeled Using Natural Language in Reinforcement Learning. https://arxiv.org/abs/1910.04040.

[38] Goyal A., Riashat I., Daniel S. (2019). Infobot: transfer and exploration via the information bottleneck. *ICLR*.

[39] Al-Shedivat M., Lee L., Salakhutdinov R., Xing E. P. (2018). On the complexity of exploration in goal-driven navigation. https://arxiv.org/abs/1811.06889.

[40] Chourasia R., Singla A. (2019). Unifying Ensemble Methods for Q-Learning via Social Choice Theory. https://arxiv.org/abs/1902.10646.

[41] Shang W., Trott A., Zheng S., Xiong C., Socher R. (2019). Learning World Graphs to Accelerate Hierarchical Reinforcement Learning. https://arxiv.org/abs/1907.00664.

[42] Goyal A., Sodhani S., Binas J., Bin Peng X., Levine S., Bengio Y. (2019). Reinforcement learning with competitive ensembles of information-constrained primitives. https://arxiv.org/abs/1906.10667.

[43] Chen D., Yan Q., Guo S., Yang Z., Su X., Chen F. (2019). *Learning Effective Subgoals with Multi-Task Hierarchical Reinforcement Learning*.

[44] Raileanu R., Rocktäschel T. (2020). RIDE: rewarding impact-driven exploration for procedurally-generated environments. *ICLR*.

[45] Campero A., Raileanu R., Tenenbaum J. B., Rocktäschel T., Grefenstette E. (2021). Learning with amigo: adversarially motivated intrinsic goals. *ICLR*.

[46] Mirchandani S., Karamcheti S., Sadigh D. (2021). ELLA: exploration through learned language abstraction. *Advances in Neural Information Processing Systems*.

[47] Zhu H., Paschalidis I. C., Hasselmo M. E. Feature extraction in Q-learning using neural networks.

[48] Phaniteja S., Dewangan P., Guhan P., Sarkar A., Krishna K. M. A deep reinforcement learning approach for dynamically stable inverse kinematics of humanoid robots.

[49] Sharma A. R., Kaushik P. Literature survey of statistical, deep and reinforcement learning in natural language processing.

[50] Kilin A., Bozek P., Karavaev Y., Klekovkin A., Shestakov V. (2017). Experimental investigations of a highly maneuverable mobile omniwheel robot. *International Journal of Advanced Robotic Systems*.

[51] Tătar M. O., Popovici C., Mândru D., Ardelean I., Pleşa A. Design and development of an autonomous omni-directional mobile robot with Mecanum wheels.

[52] Krinkin K., Stotskaya E., Stotskiy Y. Design and implementation Raspberry Pi-based omni-wheel mobile robot.

[53] Krizhevsky A., Sutskever I., Hinton G. E. (2012). ImageNet classification with deep convolutional neural networks. *Advances in Neural Information Processing Systems*.

[54] Russakovsky O., Deng J., Su H. (2015). ImageNet large Scale visual recognition Challenge. *International Journal of Computer Vision*.

[55] He K., Gkioxari G., Dollár P., Girshick R., Mask R.-C. N. N.

[56] Ren S., He K., Girshick R., Sun J., Faster R.-C. N. N. (2016). Towards real-time object detection with region proposal networks. *IEEE Transactions on Pattern Analysis and Machine Intelligence*.

[57] Redmon J., Divvala S., Girshick R., Farhadi A. You only look once: unified, real-time object detection.

[58] Girshick R. Fast R-CNN.

[59] Girshick R., Donahue J., Darrell T., Malik J. Rich feature hierarchies for accurate object detection and semantic segmentation.

[60] Tan M., Pang R., Le Q. V. Efficientdet: scalable and efficient object detection.

[61] Tan M., Le Q. V. (2020). Efficientnet: Rethinking Model Scaling for Convolutional Neural Networks. https://arxiv.org/abs/1905.11946.

[62] Moghaddam M., Charmi M., Hassanpoor H. (2021). Jointly Human Semantic Parsing and Attribute Recognition with Feature Pyramid Structure in EfficientNets. *IET Image Process*.

[63] Zhou Y., Shi J., Yang X., Wang C., Wei S., Zhang X. (2019). Rotational objects recognition and angle estimation via kernel-mapping CNN. *IEEE Access*.

[64] Elad M. (2002). On the origin of the bilateral filter and ways to improve it. *IEEE Transactions on Image Processing: A Publication of the IEEE Signal Processing Society*.

[65] Pfeiffer M., Shukla S., Turchetta M. (2018). Reinforced imitation: sample efficient deep reinforcement learning for mapless navigation by leveraging prior demonstrations. *IEEE Robotics and Automation Letters*.

[66] Hsu S.-H., Chan S.-H., Wu P.-T., Xiao K., Fu L.-C. Distributed deep reinforcement learning based indoor visual navigation.

[67] Long P., Fan T., Liao X., Liu W., Zhang H., Pan J. Towards Optimally Decentralized Multi-Robot Collision Avoidance via Deep Reinforcement Learning; towards Optimally Decentralized Multi-Robot Collision Avoidance via Deep Reinforcement Learning.

[68] Wang C., Wang J., Shen Y., Zhang X. (2019). Autonomous navigation of UAVs in large-scale complex environments: a deep reinforcement learning approach. *IEEE Transactions on Vehicular Technology*.

[69] Lin J., Yang X., Zheng P., Cheng H. (2019). End-to-end decentralized multi-robot navigation in unknown complex environments via deep reinforcement learning. *End-to-end Decentralized Multi-Robot Navigation in Unknown Complex Environments via Deep Reinforcement Learning*.

[70] Wang C., Wang J., Wang J., Zhang X. (2020). Deep-reinforcement-learning-based autonomous UAV navigation with sparse rewards. *IEEE Internet of Things Journal*.

[71] Leiva F., Ruiz-Del-Solar J. (2020). Robust RL-based map-less local planning: using 2D point clouds as observations. *IEEE Robotics and Automation Letters*.

[72] Qadir Q. M., Rashid T. A., Al-Salihi N. K., Ismael B., Kist A. A., Zhang Z. (2018). Low power wide area networks: a survey of enabling technologies, applications and interoperability needs. *IEEE Access*.

[73] Shahjalal M., Hasan M. K., Islam M. M., Alam M. M., Ahmed M. F., Jang Y. M. An overview of AI-enabled remote smart- home monitoring system using LoRa.

[74] Sartoretti G., Kerr J., Shi Y. (2019). PRIMAL: pathfinding via reinforcement and imitation multi-agent learning. *IEEE Robotics and Automation Letters*.

[75] Lin J., Yang X., Zheng P., Cheng H. End-to-end decentralized multi-robot navigation in unknown complex environments via deep reinforcement learning.

[76] Fan T., Long P., Liu W., Pan J. (2018). Fully distributed multi-robot collision avoidance via deep reinforcement learning for safe and efficient navigation in complex scenarios. https://arxiv.org/abs/2207.10417.

[77] Yahya A., Li A., Kalakrishnan M., Chebotar Y., Levine S. Collective robot reinforcement learning with distributed asynchronous guided policy search.

